# Epidemiological characteristics of crashes and pattern of motorcycle injuries presenting to hospitals in Kisumu City, Kenya: a cross-sectional study

**DOI:** 10.11604/pamj.2023.45.75.39658

**Published:** 2023-06-02

**Authors:** Wilberforce Cholo, Wilson Odero, Japheths Ogendi

**Affiliations:** 1 Department of Public Health, Maseno University, Kisumu, City Kenya and,; 2 Department of Public Health, Masinde Muliro University of Science and Technology, Kakamega, Kenya,; 3 School of Medicine, Maseno University, Kisumu City, Kenya.

**Keywords:** Motorcycle, injuries, Kisumu city, tier III hospitals

## Abstract

**Introduction:**

motorcycle injuries comprise considerable morbidity, disability and mortality of road traffic casualties. The study aimed to assess the pattern and characteristics of motorcycle crash injuries that sought care at the Emergency Departments in Tier III hospitals in Kisumu City during a six-month period from May to November 2019.

**Methods:**

this was a cross-sectional study of all motorcycle injury patients presenting to three tier III public and private hospitals in Kisumu City. Using a structured questionnaire, data were collected on human and injury characteristics. Multiple logistic regression model was used to determine the predictors of fatality. Statistical significance was set at p<0.05.

**Results:**

a total of 1073 of motorcycle crash injury patients sought and obtained care at the hospitals. The majority (73.6%) were males. Seventy-three cases resulted in death (case-fatality rate of 6.80%. The age range was 2-84 years, with a mean of 29.6 years (± SD 12.19). Majority, (43.3%) were aged 21-30 years. Most of the crashes occurred during the daytime (79.1%). Of all motorcycle riders and pillion passengers 30.6% wore helmets at the time of the crash. Head injuries (43.6%) were the most common. Injury seventy scores (ISS) ranged from 1 to 51. Glasgow comma scale <3, un-helmeted patients and major trauma (ISS > 16), significantly influenced mortality (P< 0.001).

**Conclusion:**

these findings confirmed and strengthened the documented substantial morbidity, mortality that motorcycle crashes place on individuals and society, contribute to the body of literature on motorcycle injuries and potentially assist in policy decisions on motorcycle transport safety.

## Introduction

Every year, road traffic crashes cause over 1.36 million deaths and 20-50 million injuries that result in hospital visits globally [1]. Road traffic crashes are the eighth leading cause of death for people of all ages and the most important cause of death in children and young adults, 5-29 years of age [1]. In 2015 road traffic injuries were the 9^th^ leading cause of disability adjusted life years (DALYS) but are projected to be the fourth leading cause of disease burden by 2030 [2]. The burden of road traffic injuries is borne disproportionately by the low- and middle-income countries (LMICs) with nearly 85% of deaths and 90% of DALYs lost [1]. In low income countries (LIC), road traffic injuries constitute a major but neglected public health problem and impact negatively on the economy and health services [3]. The road traffic death rate in low-income countries is three times higher than in high income countries [1]. Sub-Saharan Africa has the highest road traffic death rate (26 deaths per 100,000 populations) three times higher than Europe (9.3 deaths per 100,000 populations [1]. The rate of fatalities is even higher at 34.4 per 100000 persons in Kenya [4]. Globally, vulnerable road users comprise 54% of all road deaths consisting of pedestrians (23%), bicyclists (3%), and 28% occur among motorcyclists, [1,5] and in the World Health Organization (WHO) Africa region 7 to 16% of all road traffic deaths are motorcycle related [1,3]. This makes them an important group of road users to target for reduction of road traffic injuries [6]. The risk of dying from a motorcycle crash is 34 times higher than from a motor vehicle crash [6,7]. Factors fuelling motorcycle injuries include over-speeding, over-loading for quick returns, recklessness, and lack of respect for other road users, non-use of helmets and alcohol consumption [8,9]. Other studies have reported males, younger age and driving experience as other factors [8,10]. In addition to these behavioral factors, there are those related to the condition of the roads or the condition of the vehicles. Despite, motorcycle as means of transport is patronized because they are more affordable and flexible than cars and provide an easier way of navigating urban traffic, and they are available throughout the day and night hours [11].

Previous studies demonstrated that 98% of crashes are preventable [12]. Measures to reduce road carnage include road quality improvements, speed bumps and speed limits [13]. However, due to inadequate funding, infrastructural improvements are not attractive despite their relative cost-effectiveness; improving personal safety and enforcement of applicable rules, particularly helmet use and interventions that reduce alcohol use while riding and driving are essential. Previous studies demonstrated effectiveness of helmets in reducing death by 42% and head injury by 69% in road traffic injuries (RTIs) [9]. Unfortunately, there is poor helmet usage among motorcycle users in Kenya [14]. In East African countries, studies have shown that 58.8% of road traffic injuries are attributed to motorcycle injuries resulting into high comorbidities, fatalities and disability [15]. In Kenya, road traffic crashes kill over 3,000 people annually with an estimated cost of 4 billion Kenya shillings [16,17]. Road traffic death rate in Kenya is 34.4 per 100000, approximately 4 times higher than the high Income Countries [4]. The proportion of motorcycle as mode of transport in the country has increased 26 folds, from 53,508, in 2004 to 1,393,390 in 2018 and was accompanied by a 29.9% increase in deaths in this category of road users [18]. Motorcycle injuries is a growing public health concern in Kenya because of increasing number of motorcycle crash deaths in Kenya in recent years. For example, between the year 2005 and 2021, the reported motorcycle crash deaths, as a proportion of total reported road traffic deaths, rose from 6% to 37.6% respectively [18], over six-fold increase in just 16 years. This could be attributed to over-speeding, recklessness by the riders, alcohol use and poor use of helmets in Kenya. In addition, motorcycle riders and pedestrians are most affected road users by motorcycle crashes [10,14]. In Kisumu City, motorcycle crashes account for between 41% and 62% of road traffic injuries respectively [13, 19]. The Kisumu County government has put in place road safety intervention strategies in its plan [19]. However, Kisumu City has limited data on the epidemiological characteristics and pattern of motorcycle crash injuries that would inform context-based and data-driven safety planning and intervention strategies. The few available studies have been population based [20-22]. Previous hospital-based studies have been limited in scope by being set in only single facility (13). No studies have been conducted in more than one hospital. Furthermore, no studies have focused on injuries and mortality estimates due to motorcycle injuries simultaneously. This study assessed and documented the magnitude, epidemiological characteristics and pattern of motorcycle crash injuries that obtained care at the Emergency Departments of Tier III hospitals in Kisumu city during a six-month period. Documenting the epidemiological characteristics of motorcycle injuries is crucial for policy decisions and actions to reduce the consequences of motorcycle crash injuries and increase understanding of their impact.

## Methods

**Study design:** this was a cross-sectional study in which a quantitative data collection strategy was used to gather information. Consecutive trauma patients presenting to the hospitals, over a six-month period (between May 6, 2019 and November 6, 2019) were recruited prospectively.

**Study setting:** this study was conducted in hospitals graded as Tier III Hospitals in Kisumu City over a period of 6 months from 6^th^ May to 6^th^ November 2019. Kisumu City is the third-largest city after Nairobi and Mombasa [23]. The city has a population of 610,082 persons [23]. Health services in the city are provided by public and private sectors [24,25]. The study focused on the three hospitals, namely, Jaramogi Oginga Odinga Teaching and Referral Hospital, Kisumu County Hospital and Aga Khan Hospital, ranked in Tier III [25]. Jaramogi Oginga Odinga Teaching and Referral Hospital (JOOTRH), Kisumu County Hospital are public hospitals run by Kisumu County government [21]. Aga Khan Hospital is privately run. These hospitals were selected because they all have active emergency departments that provide care to the crash patients and operate on 24 hour basis [24]. The main mode of transportation used within the Kisumu City is walking which comprise slightly more than one half (53%) of daily trips [20,25]. Motorcycle, matatus and bicycles comprise (19%), (13%) and (4%), of urban transport respectively [25].

**Study participants:** all motorcycle crash injury patients that obtained care at the Accident and Emergency Departments of Tier III hospitals in Kisumu City, namely, Jaramogi Oginga Odinga Teaching and Referral Hospital, Kisumu County Hospital and Aga Khan Hospital during a six-month period, from May 6, through to November 6, 2019.

**Sample size determination:** sample size (n) was computed as a function of 95% power level (1-β), 5% (α- 0.05), significance level and 10% effect size using a priori power analysis. A priori analysis is an effectual method in controlling statistical power before a study is actually executed [26]. Based on this method the sample size was 1073 participants.

**Sampling methods and procedure:** total population sampling method was used to successively enroll all motorcycle injury patients that presented in the Emergency Departments of the Tier III hospitals for a period of six months. This type of sampling provides a much more representative assessment than the alternative methods [27].

**Data sources, collection and instrument:** data were gathered on all motorcycle injury patients who presented to the Emergency Department of Tier III hospitals over a period of six months(from 6^th^ May, 6^th^ November, 2019). Patients were assessed according to advanced trauma life support guidelines and contemporary standards of trauma care. Consecutive trauma patients presenting to the hospitals, over a six-month period were recruited. Motorcycle crash injured patients gave their informed consent either after they were stabilized or relatives who brought them for care assented. Data were collected by trained research assistants who completed a pretested structured questionnaire.

**Variables and measurements:** the variables captured were: epidemiological characteristics including; sociodemographic, time, behavioral (helmet use, reflective clothing, alcohol consumption) and injury characteristics; injury disposition, length of stay (LOS), injury location, diagnosis and severity. Injury diagnosis was measured through clinical history, examination and radiological investigations. Severity of injury sustained was assessed and measured by the attending physician using the Abbreviated injury scale (AIS) and Injury severity score (ISS) scale on the basis of the clinical diagnosis. The ISS was measured using the Abbreviated injury scales (AIS) updated version [28], as documented by Baker *et al*. [29]. The ISS ranged from 0 to 75 [29], and were categorized in four groups: ISS 0-8, 9-15, 16-24 and 24+. Further classification was done into minor (ISS <16) and major injuries (ISS≥16) [30]. Glasgow comma scale (GCS) was used to measure physiological characteristics and categorized into mild, moderate and severe. Injury outcome were captured as discharged alive, referred or death. Motorcycle fatality data were obtained from the postmortem reports. The person in charge of each mortuary within the hospitals was requested to assist in gathering mortality related data.

**Data analysis:** the quantitative data were coded and entered into SPSS version 24 program [31]. Descriptive statistics were used to generate frequency distribution of sociodemographic, behavioral and injury characteristics. Chi square was used to analyze the association between helmet use and sex; anatomic injury site. Logistic regression analysis was used to estimate association (crude odd ratio) between fatality and demographic, injury and behavioral characteristics(p-value < 0.05). Further, a multivariable logistic regression model was used to compute adjusted odds ratios with 95% CI, and P- value <0.05% considered statistically significant.

**Ethical and permission clearance:** research and ethical approvals were sought and obtained from the Maseno University School of Graduate Studies, Ethical clearance were sought and obtained from Maseno University Ethics Review Committee (MUERC) and Jaramogi Oginga Odinga Teaching and Referral Hospital Ethical Review Committee. Permission and authority were sought and obtained from National Commission for Science, Technology & Innovation (NACOSTI). Permission was also sought from the County Health Director, Administrators from the three Tier III hospitals in Kisumu City. Consent was received from all the participants before enrollment.

## Results

**Figure 1:**
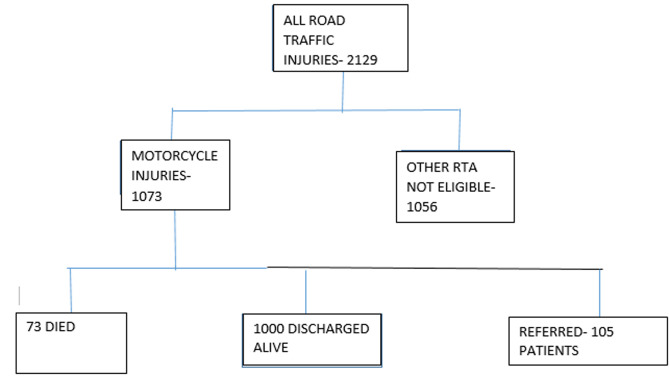
flow chart showing enrolment of eligible motorcycle injury patients

**Participants:** during the 6-month period between May and November 2019, there were 2129 road traffic injury cases presenting for treatment to the hospitals in Kisumu City. Of these, 1073 (50.4%) were motorcycle injury cases. Of the 1073 motorcycle injury cases, all met study criteria and were enrolled. The study was designed to enroll 1073 participants, all the eligible participants/ or caregivers consented of which 6.8% died (Figure 1).

**Demographic characteristics of motorcycle injury patients:** of all the motorcycle injury patients 790 (73.6%) were male (M: F ratio= 2.8: 1). The ages ranged from 2 years to 84 years with mean age of 29.6 (± SD 12.19). The majority (43.3%) were in the age of 21-30 years; followed by 249 (23.2%) in the age bracket of 31-40 years. An overwhelming majority of 986 (91.9%) were those in the economically active age bracket of between 20 and 49 years. Majority 516 (48.1%) of the motorcycle injury cases had attained secondary education. More than half 683 (63.7%) of the motorcycle injured victims were motorcycle riders 363 (33.8%) and their pillion passengers 321 (29.9%). About a quarter of motorcycle injury cases 293 (27.3%) had an average income of less than Kshs. 10, 000 while 227 (21.3%) of the casualties had no income as indicated in Table 1.

**Table 1:** sociodemographic characteristics of motorcycle injuries

Characteristic	Frequency (Percent %)
**Gender**	
Male	790 (73.6)
Female	283 (26.4)
**Age group (yrs.)** 0-10	67(6.2)
11-20	113(10.5)
21-30	464 (43.3)
31-40	249(23.2)
41-50	94(8.8)
>50	86(8.0)
**Level of education** Primary	271(25.3)
Secondary	516(48.1)
Tertiary college	245(22.8)
University	10(0.9)
None	31(2.9)
**Average income** Less than 10000	293(27.3)
10000-20000	25(2.3)
21000-30000	342 (31.9)
41000-50000	41(3.8)
51000-60000	131(12.2)
Above 50000	14(1.3)
None	227(21.2)
**Road user category**	
Motorcycle rider	363(33.8)
Passenger	321(29.9)
Pedestrian	373(34.8)
Bicyclist	16(1.5)
Total	1073(100)

**Pre hospital care, transportation and time taken to the hospital:** fifty-three percent of the motorcycle injury patients (570) received some form of prehospital care. There were about 2.9% (31) motorcycle pre-hospital fatalities. However, Nearly 92% of the motorcycle crash injury patients were transported to hospital by either relatives, self or bystanders. Only 6.8% were transported by ambulance. Nearly half of the motorcycle injury cases 531 (49.5%) reported to the hospitals within 1-6 hours after the crash.

**Behavioral safety practices of motorcycle injury cases:** the study found that the proportion of protective behavior among motorcycle crash cases was low. Of the 684 passengers and motorcycle riders, almost seventy percent 474 (69.3%) did not use helmets at the time of the crash. Approximately, 353 (32.9%) of all motorcycle injury cases admitted having consumed alcohol before the crash. Among the un-helmeted, the majority of the males sustained head and neck injury >291( 48.1%). However, males with helmets had fewer head and facial injuries (p>0.001). Overall, helmet use among female passengers was low at 21(1.95%). There was increased proportion of severe injuries (ISS >16) on the head and neck region 75 (55.1%) among the un-helmeted (P=0.001), and multiple site injuries (P=0.001). Even with less severe injuries (ISS<16), nearly half of 176 (49.2%) of motorcycle riders and pillion passengers who sustained head and neck injuries did not wear helmets. Almost three quarters 70.1% of the motorcycle riders and their pillion passengers who sustained severe injuries (ISS>16) were un-helmeted. Only 29.2% of motorcycle injuries patients used reflective jackets.

**Distribution of motorcycle injuries by mechanism of occurrence:** overall, motorcycle crashes have different characteristics due to collisions involving other road user groups. Compared to other causes, motorcycle-pedestrian and motorcycle-motorcycle were the commonest cause of crashes representing 328 (30.6%) and 271(25.3%) respectively.

**Distribution of motorcycle injuries among road users by time and day of the week of the crash:** as shown in Table 2 most crashes occurred between 6 pm-9pm and 6-9 am accounting for 27.1%, and 26.7% respectively. The highest proportion of crashes occurred on Monday 277 (25.8%). Areas close to the urban centers contribute the highest burden of the crashes in 918 (85.6%). The distribution of motorcycle related injuries by day of occurrence and road-user involved showed that pedestrian and motorcycle riders were significantly more likely to be involved in weekdays crashes (OR=3.8; Cl: 2.7-5.3, p<0.003) and (OR=3.6; Cl: 2.2- 4.6, p<0.003) respectively. Majority (40.5%) sustained motorcycle crash injuries on Mondays and Fridays. When the time of motorcycle injury occurrence was taken into account, a greater proportion of motorcycle riders (75.6 %%) were found to have been involved in crash during the day between 6am- 5pm in comparison to pedestrians (68.6%) and bicyclists (56.3%). The probability of crashing at night between 6.00-9.00pm was three times greater for pedestrian when compared to bicyclists (OR=2.9; Cl: 1.1- 6.3, p<0.02). There was a significant association between the type of road user and the day of the week a crash occurred (p <0.001).

**Table 2:** distribution of injuries among road user by time and day of the crash (n=1073)

Road user	Motorcycle rider	Passenger	Pedestrian	Bicyclist	Total	P-value
**Time of the crash**						
6-9am	117(33.3)	77(24.0)	90(24.1)	2 (12.5)	286 (26.7)	0.02
10-1pm	59( 16.1)	63(19.6)	88(23.6)	1 (6.3)	211 (19.7)	
2-5pm	99(26.0)	85 (26.5)	78(20.9)	6 (37.5)	268 (25.0)	
6-9pm	76 (21.1)	87 (27.1)	102 (27.3)	6( 37.5)	271(25.3)	
10pm-1am	10(2.9)	7 (2.2)	15(4.0)	1 (6.3)	33(3.1)	
2-am-5am	2 (0.6)	2 (0.6)	0 (0.0)	0(0.0)	4 (0.4)	
**Day of the crash**						
Monday	92 (22.20)	80(23.40)	102 (24.70)	1(6.20)	277 (25.8)	<0.0001
Tuesday	37(10.20)	46 (14.30)	39(10.50)	2 (12.50)	124 (11.6)	
Wednesday	22 (7.30)	24 (7.50)	35(9.40)	1(6.20)	87 (8.10)	
Thursday	47(11.10)	36 (11.20)	40 (10.70)	0(0.00)	123 (11.50)	
Friday	73(18.10)	52(16.20)	98 (23.60)	6 (37.50)	229 (21.3)	
Saturday	72 (19.90)	60 (18.70)	35 (9.40)	1(6.20)	168 (15.70)	
Sunday	18 (11.10)	23(8.70)	24 (11.80)	5 (31.20)	65 (6.1)	

**Anatomical site or body region affected by motorcycle injury:** the distribution of nature of injuries was assessed according to classification method presented by Baker *et al*. [29], head -neck and Lower extremity injuries of the patients were the most sustained injuries representing 468 (43.6%) and 214 (19.9%) of the patients respectively. Upper extremity injuries affected 7.9% of the patients while 125 (11.6%) had variable chest injuries, including rib fractures and various forms of abrasions and lacerations including superficial and lung and heart lacerations. A total of 958 (88.4.6%) patients had isolated trauma to one system, while the remainder 115 (11.6%) had multisystem trauma. The most common pattern of injury in poly traumatized patients was combined head and orthopedic injuries in 40 (35.2%) of patients. Injury to the head and maxillofacial and abdomen and orthopedic accounted for 20 (18%) and 18 (16.0%) respectively while injury to the head, chest, abdomen and orthopedic comprised of 6 (4.8%).

**Injury severity:** injury seventy scores (ISS) ranged from 1 to 51. The overall mean ISS was 12.9, with a median of 10.7. Majority 815 (73.0%) of the motorcycle injury cases sustained mild -moderate injuries (ISS<16). Severe injuries, (ISS >16), occurred in more than a quarter (27.0 %) of the cases, with a mean ISS of 24.6 (range 16-51). Of the overall motorcycle injury cases that presented to the hospitals, 494 (46%) were admitted in the surgical wards. The length of stay (LOS) in hospitals ranged from 1 to 235 days with a mean of 19.8 days SD of 8.23. Fifty-two (4.9%) of the cases were admitted in the ICU; of these, 39 (73.0%) demanded ventilator support.

**Association between road user category and injury severity (ISS) and injury disposition:** most of the motorcycle injury cases (52.0%) sustained moderate injuries followed by 21.0% who had minor injuries as shown in Table 3. The proportion by ISS varied by category of road user. Approximately a half (44.5%) of all minor injuries (ISS, 0-8) were sustained by motorcycle riders, and nearly three-quarters (73.0%) of all motorcycle injuries were classified as minor or moderate. Motorcycle riders also contributed to 32 (46.4%) of all fatal injuries and 25 (51.0%) of critical injuries. Nearly three quarters of 66 (74.2%) of all serious injuries were sustained by motorcycle riders and their pillion passengers. Further analysis using regression analysis showed that when the proportion of motorcycle injury patients with ISS of less than 16 was compared to those having ISS ≥16, by road user, motorcycle riders were significantly over-represented among the severely injured group (OR=5.5; Cl: 2.4-9.2; p<0.001). Patient disposition is mirrored in outcome patterns and discharge status of the injury cases which, 251 (73.4%) of the motorcycle riders were treated and discharged routinely, 333 (89.0%) of the pedestrians were treated and discharged and 11 (2.9%) died. There were significant associations between the severity of injury as measured by injury severity score (ISS) score and type of road user (p=0.019), and between injury outcome and type of road user (p<0.0001).

**Table 3:** association between road user category and injury severity (ISS) and injury disposition (n=1073)

Road user	Motorcycle rider	Pillion passenger	Pedestrian	Bicyclist	Total	P-value
**Injury severity score (ISS)**						
0-8 (Minor)	70(19.0)	54(17.0)	99 (27.0)	1(6.0)	224(21.0)	<0.0001
8-15 (Moderate)	171 (48.0)	193(60.0)	189(51.0)	7 (44.0)	560 (52.0)	
16-24 (Serious)	38(11.0)	23(7.0)	28 (8.0)	0(0.0)	89(8.0)	
>24 (Severe/critical)	84 (22.0)	51(16.0)	57(15.0)	8(50.0)	200(19.0)	
**Injury outcome(disposition)**						
Routine discharged	274(74.8)	280(87.2)	333 (89.0)	8(50.0)	895 (83.4)	<0.0001
Referred	51(15.8)	23(7.2)	24 (6.4)	7 (43.8)	105 (10.2)	
Died	37 (9.3)	18 (5.6)	17(4.2)	1 (6.2	73 (3.4)	

**Estimation of mortality risk caused by motorcycle crashes based on sex, age, type of road user, length of stay, anatomic injury site, injury severity (ISS), and Glasgow coma scale (GCS):** the final multivariable logistic regression model indicates the predictors of fatality as presented in Table 4. The risk of fatality were significantly associated with being male, GCS, ISS, length of stay, age and sex. Males and age of 30-40 years were 3 times and 2.9 times (AOR =3.17; CI: 1.95-6.38) and (AOR=2.89; 95% CI: 0.28-7.13) more likely to die than females and patients aged 50 and above respectively. Similarly, being hospitalized for 29-42 days and sustaining head injuries had 5 times 3.5 times had increased risk of (AOR=5.23; 95% CI: 2.83-20.15) and (AOR=3.46; 95% CI: 0.74-7.15) dying than those who were not admitted and sustaining upper extremities injuries respectively. Patients with lower GCS≤3 and High (ISS≥16) were 32 and 6 times more likely (AOR=32.45; 95% Cl: 2.24-63.95), and (AOR=6.45; 95% Cl: 2.24-13.95) to die than those with GCS≤3 and ISS≥16 respectively. Other factors were being motorcycle rider (AOR=2.42; 95%CI: 0.17-6.67) and not wearing helmets (AOR=4.8; CI: 2.36-12.70).

**Table 4:** estimation of mortality risk caused by motorcycle crashes based on sex, age, type of road user, length of stay, anatomic injury site, injury severity (ISS)

Gender	Crude odds ratio (95% Cl)	P- value	Adjusted odd ratio (95% cl)	P- value
Males	3.5(3.149-7.47)	0.001	3.17(1.95-6.38)	0.001
Females	Ref		Ref	Ref
**Age**				
<18	Ref		Ref	
19-30	1.46(0.66-3.8)	0.03	0.91(0.18-1.92)	0.046
31-40	2.72(0.54-5.38)	0.006	2.89(0.28-7.13)	0.002
41-50	1.0(0.10-2.77)	0.34	0.56(0.16-1.16)	0.42
above 50	1.0(0.83-1.26)	0.32	0.93(0.36-1.54)	0.1
**Type of road user**				
Motorcycle rider passengers pedestrian bicyclist	1.4(0.604-10.176) 0.62(0.477-3.364) 0.77( 0.193-3.999) ref	0.008 0.25 0.22 Ref	2.42(0.17-6.67) 1.06(0.74-2.02) 0.98(0.14-2.06) Ref	0.03 0.9 0.6 Ref
**Length of stay**			;	
1-6	1.0(0.09-1.93)	0.56		
7-14	1.9(0.74-2.15)	0.21	2.16(0.74-4.15)	0.26
15-28	2.48(0.74-1.85)	0.06	3.2(0.63-8.85)	0.36
29-42	2.3(0.93-4.14)	0.001	5.23(0.83-20.15)	0.02
43-56	3.82(0.58-18.6)	0.13	1.18(0.58-2.40)	0.01
Above 57	14.4 (3.85–23.68)	0.01	2.45(1.18-5.10)	0.79
N/A	ref			
**Helmet use**				
Yes	1.5(1.16-3.67)	0.17	1.6(0.81-3.34)	0.08
No	4.94 (3.3-10.29)	<0.001	5.(0.36-12.70)	<0.001
N/A	ref		Ref	
**Anatomic injury site**				
Head, face and neck	5.07(1.46-20.67)	<0.001	3.46(0.74-7.15)	<0.001
Chest/thorax	0.42(0.23-1.15)	0.008	0.61(0.13-1.35)	0.08
Abdomen	0.59(1.65-6.67)	0.09	1.01(0.14-3.15)	0.52
Lower extremities	0.28(1.3-15.08)	0.25	0.45(0.24-0.95)	0.02
Upper extremities	ref	ref	ref	
**Injury severity (ISS)**				
ISS≤16	Ref		ref	ref
ISS≥16	3.20(1.25-14.67)	0.01	6.45(2.24-13.95)	<0.001
**Glasgow Coma Scale(GSC)**				
≤3	13.50(1.45-77.67)	0.01	32.45(2.24-63.95)	<0.001
≥3	Ref	ref	ref	
**Pre hospital care obtained**				
Yes	0.82(0.08-1.58)	0.9	0.96(0.48-1.08)	
No	4.10(1.25-18.67)	0.004	4.10(1.25-22.62)	0.001
N/A	ref		ref	ref

## Discussion

Road transportation plays an important part in a society for the movement of people and goods. The attendant consequences of road crashes cannot be overemphasized as it leads to morbidity, mortality, disability and increased economic cost in terms of managing injuries and hospitalization. Despite the high burden, there has been low targeted public policy responses to address the rising problem because of inadequate reliable data on motorcycle road safety. The study found that motorcycle injuries are more prevalent than other RTA in Kisumu City, and are responsible for over half of RTA-related injuries and deaths. Death among all motorcycle injury victims was significantly higher among those aged 31 to 40 years, and those with low Glasgow Coma Scores. The demographic profile of those injured motorcycle users was similar to the general road traffic injuries. In consistent to other studies, the majority of people of productive age group were involved in motorcycle crashes [32,33]. The young adults are most involved in motorcycle crashes with mean age of 29.83 years ± SD 12.19 with a peak in the 21- 30 years group. This age-group is the most productive age group, and thus, probably indicates that health promotion strategies on road safety should target this same group. Males outnumbered females with the proportions of male motorcycle injury and fatality being as high as 73.6% and 90.1% respectively, and if current trend continues, more adult young men in the economically productive group will continue to die or get injured as a result of motorcycle crashes, thus reducing productivity. The young male preponderance in this study concurs with findings reported in other settings [6,11,34]. This has been attributed to a number of reasons; namely, they are more likely to engage in high risk activities such as overloading, reckless riding, over speeding, adventurous, and alcohol use. In many occasions males travel for longer distances. In agreement with previous studies, majority of motorcycle crash injuries and fatalities occurred among motorcycle riders and pedestrians [34,35]. Motorcyclists are particularly vulnerable to injury, as they do not have the protective steel car frame to absorb the transmitted forces imparted during a collision. There is a massive amount of energy transferred to the motorcyclists upon impact. Most of them over speed and ride under the influence of alcohol increasing their risk of crash.

The analysis of accidents based on time of occurrence pointed out that most motorcycle crashes happened during the day. The study finding agrees with studies in Tanzania [12] in Ghana [7,36] in UK [37], and, in Iran [38]. Increased proportion of injuries during the day can be explained by increased traffic density as well as increased human activities in the City during the daytime. Knowing the time of injury in trauma patients is important for prevention strategies and resource allocation. Majority of the motorcycle crash injury patients (49.5%) arrived at the hospitals within 1-6 hours after the crash. This is in agreement with other road safety studies [12,16]. The delay in seeking medical care is likely to contribute significantly to morbidity and mortality among trauma patients [1]. The pre-hospital care of a trauma patient has been reported to be the most important determinant in the ultimate outcome after the injury [6,39]. Among the motorcycle injury cases, 53.1% were given some form of pre-hospital care. Previous studies in Rwanda, and Tanzania indicated that motorcycle injury cases were brought in by relatives and police who are not trained in healthcare or patient transport [12,40,41]. Prompt effective, rapid and competent pre-hospital care reduces significantly the consequences of crashes. According Gunchan, Gautam, and Rubina outcomes of injuries are affected by pre-hospital care and condition of the patient on arrival at the emergency department [42]. The motorcycle crash injuries ranged from minor abrasions to poly-trauma cases and the life-threatening head injuries and fractures. Motorcycle injury studies in developing countries have shown that head injuries and musculoskeletal injuries are the most common causes of morbidity and mortality in motorcycle injuries [6,43]. Injuries to the head and extremities accounted for most cases, and was consistent with findings in studies elsewhere [16,40,43,44]. Further, 74.2% of all admitted cases had head Injuries and injuries to extremities. This was comparable to studies conducted in Rwanda [44], Uganda [45], United Kingdom [46], and Nigeria [47]. Injuries to the lower extremities were more on motorcycle riders and pedestrians. Patterns of injury risk suggest that the lower limbs are the body parts most likely to be injured in motorcycle crashes particularly among the motorcyclist and pedestrians. The susceptibility of lower limbs is due anatomical location and lack of protection on the extremity since the limbs are often squeezed between the motorcycle and impacting vehicle, the ground or some other fixed object. Interventions to prevent lower limb injury such as wearing protective boots, knee pads, and padded gears should be emphasized.

Injury severity score provides a valid measure of morbidity status of a casualty and the prognosis of survival or death from multiple injuries [29]. Injury severity score remains the most widely used method for determining overall injury severity [29]. Patients with low ISS have less forms of injuries, and generally, recover fast, while those with greater scores are more likely to have a longer period of recuperation. In this study, most patients (72%) presented with injuries of minor and moderate character (ISS 0-16), with nearly over 27% of participants sustaining a severe injury in a motorcycle crash. Previous studies globally have reported that use of helmets by both the motorcyclists and their pillion passengers significantly reduce the incidence of fatal head injury [48]. However, the use of crash helmets is still very low in Kenya [14]. Helmet use among motorcycle riders and their pillion passengers varies across developing countries [48,49]. Overall, in this study the prevalence of helmet use was 30.7% which was far much lesser than 56%, 51.4%, 72.8%, 82.8% and 99 % reported in Nigeria [50], Jamaica [51], China, [52], Spain [53], and Vietnam [54] and was higher than 4% in Ghana [55] respectively. These variations in the proportions of helmet use show an impression on the differences in levels of awareness on injury occurrence, their severity and associated factors between these countries and poor enforcement of traffic laws. Rice *et al*., found that the risk of sustaining a moderate to severe head injury by not wearing a helmet was 5 times higher than had a helmet been worn [56]. In this study, 84.5% of the fatal cases did not wear helmets at the time of the crash and were 5 times more likely to die. Almost three quarters 70.1% of the motorcycle riders and their pillion passengers who sustained severe injuries (ISS>16) were un-helmeted. The essentially of helmet use is compelling in this study. The effectiveness of Helmet laws in increasing use of helmets by motorcycle riders and passengers and reducing head injuries has been shown in other studies [8,10,47,57]. In Kenya, mandatory helmet law was enacted in 2009 and 2012 in the Traffic Act in 2012 [58,59]. But the wearing rate remains low because of non-compliance and reluctant enforcement by the authorities. There is need for strict enforcement and increased publicity of the law to improve compliance. These results should be considered in light of a study limitation. This study focused only on motorcycle injury patients who sought and obtained care in 3 major referral hospitals, and nothing is known about those seeking care elsewhere. Therefore, motorcycle injury cases are likely to be underestimated. Nevertheless, useful data on the epidemiological characteristics of injured patients seeking care in hospitals could be obtained since all cases were enrolled. Enrolling all cases has been documented to provide a much more representative of the burden of motorcycle crashes and provide greater statistical power; thus the results could be generalized for studies conducted in referral hospitals.

## Conclusion

Motorcycle crash injuries form an important but neglected public health problem in Kisumu City. The young adult males in their productive age group are commonly affected. Injuries to the head and extremities are the most common types of motorcycle injury sustained leading to hospitalization. Motorcycle injury victims with a lower GCS score and a higher ISS score, who sustained head injuries and did not wear helmets had increased risk of fatality. Since the majority of motorcycle injuries are preventable, there is need for stronger enforcement and increased publicity and application of road traffic safety laws and regulation to improve compliance.

### What is known about this topic

- *Increased use of motorcycle for public transport has been accompanied by increased road traffic injuries and deaths*;
- *Motorcycle injuries and their pillion passengers sustain severe injuries to the head and extremities*;
- *Paucity of epidemiological data on motorcycle injuries in Kisumu City, Kenya*.

### What this study adds

- *Provides the magnitude and epidemiological pattern characteristics of motorcycle injuries*
- *Documents a comprehensive summary of predictive factors of VF among pediatric patients in sub-Saharan Africa*.
